# In Vivo Microbeam Radiation Therapy at a Conventional Small Animal Irradiator

**DOI:** 10.3390/cancers16030581

**Published:** 2024-01-30

**Authors:** Mabroor Ahmed, Sandra Bicher, Stephanie Elisabeth Combs, Rainer Lindner, Susanne Raulefs, Thomas E. Schmid, Suzana Spasova, Jessica Stolz, Jan Jakob Wilkens, Johanna Winter, Stefan Bartzsch

**Affiliations:** 1 Department of Radiation Oncology, School of Medicine and Klinikum rechts der Isar, Technical University of Munich, 81675 Munich, Germany; mabroor.ahmed@helmholtz-munich.de (M.A.); sandra.bicher@tum.de (S.B.); stephanie.combs@tum.de (S.E.C.); susanne.raulefs@helmholtz-munich.de (S.R.); thomas.schmid@helmholtz-munich.de (T.E.S.); suzana.spasova@tum.de (S.S.); jessica.stolz@helmholtz-munich.de (J.S.); wilkens@tum.de (J.J.W.); johanna.winter@helmholtz-munich.de (J.W.); 2 Helmholtz Zentrum München GmbH, German Research Center for Environmental Health, Institute of Radiation Medicine, 85764 Neuherberg, Germany; rainer.lindner@helmholtz-munich.de; 3 Department of Physics, School of Natural Sciences, Technical University of Munich, 85748 Garching, Germany; 4 Heinz Maier-Leibnitz Zentrum (MLZ), 85748 Garching, Germany

**Keywords:** microbeam radiation therapy, spatially fractionated radiation therapy, treatment planning, dose calculation, dosimetry, small animal irradiator, histology

## Abstract

**Simple Summary:**

Microbeam radiation therapy (MRT) is a novel, still pre-clinical form of radiation therapy for cancer treatment, where the dose is applied in spatial fractions. MRT was shown to be able to treat tumors effectively while causing reduced damage to normal tissue. Research on MRT nowadays requires large, expensive, and difficult-to-access facilities. In this study, we aim to develop an easily accessible MRT setup utilizing a conventional small animal irradiator. We developed a comprehensive treatment planning system with a dose calculation accuracy of 10%. We successfully applied microbeam radiation to a mouse in vivo and showed that the microbeam pattern is preserved by analyzing histological sections of a mouse brain. We demonstrated the feasibility of MRT using our developed setup.

**Abstract:**

Microbeam radiation therapy (MRT) is a still pre-clinical form of spatially fractionated radiotherapy, which uses an array of micrometer-wide, planar beams of X-ray radiation. The dose modulation in MRT has proven effective in the treatment of tumors while being well tolerated by normal tissue. Research on understanding the underlying biological mechanisms mostly requires large third-generation synchrotrons. In this study, we aimed to develop a preclinical treatment environment that would allow MRT independent of synchrotrons. We built a compact microbeam setup for pre-clinical experiments within a small animal irradiator and present in vivo MRT application, including treatment planning, dosimetry, and animal positioning. The brain of an immobilized mouse was treated with MRT, excised, and immunohistochemically stained against γ H2AX for DNA double-strand breaks. We developed a comprehensive treatment planning system by adjusting an existing dose calculation algorithm to our setup and attaching it to the open-source software 3D-Slicer. Predicted doses in treatment planning agreed within 10% with film dosimetry readings. We demonstrated the feasibility of MRT exposures in vivo at a compact source and showed that the microbeam pattern is observable in histological sections of a mouse brain. The platform developed in this study will be used for pre-clinical research of MRT.

## 1. Introduction

According to the World Health Organization, about 50% of all cancer patients require radiation treatment during the course of their cancer care [1]. Despite modern techniques, including image guidance and radiotherapy approaches with protons and ions, radiation-induced acute and late side effects remain the dose-limiting factors [2]. Specifically, the treatment of radio-resistant tumors and tumors close to sensitive structures, e.g., the central nervous system, is limited since the dose required to control the tumor might lead to a non-tolerable dose deposition in healthy tissue.

An innovative, alternative radiotherapy approach is microbeam radiation therapy (MRT), a still pre-clinical treatment method that employs a spatially fractionated radiation field instead of conventional homogeneous fields. A microbeam field consists of thin micrometer-sized planar beams, which are separated by a center-to-center (ctc) distance of a few 100 μ m , leading to high-dose peak regions and low-dose valley regions in the target. This type of dose delivery has not only shown to be tolerated by normal tissue [3,4,5,6,7,8,9,10] but has also simultaneously proven the ability to delay tumor growth or even control tumors [11,12,13,14,15,16,17]. Bouchet et al. were able to show increased survival of rats bearing 9L intracranial glioma when irradiated with 400 Gy microbeams in the peak compared to uniformly applied doses [18]. Slaktin et al. showed that rat brain tissue exhibited no signs of necrosis for microbeam doses up to 5000 Gy [19]. Both studies indicate a wider therapeutic window for MRT compared to conventional radiation treatment.

However, the clinical transition of MRT is inhibited by two key factors. Due to a multitude of variable parameters in microbeam fields, an unequivocal set of parameters for treatment has not yet been identified. Furthermore, an extensive comprehension of the underlying biological mechanisms, which would allow to exploit the full potential of MRT and the development of clear treatment strategies, is still lacking.

These open questions are primarily investigated using 3rd generation synchrotrons, such as the European Synchrotron Radiation Facility in Grenoble, France. However, synchrotrons are large and expensive, which limits their accessibility and slows down progress in MRT research and clinical translation. Therefore, in this study, we aim to develop a setup that facilitates pre-clinical research with MRT in a laboratory environment by utilizing the small animal irradiator XenX (Xstrahl, Suwanee, GA, USA) [20].

Here, we present for the first time in vivo MRT treatments in a small animal irradiator. We established a treatment protocol, including treatment planning, positioning, and immobilization of the animals. The treatment planning system is based on an existing dose calculation engine for quasi-parallel synchrotron microbeams, which was adjusted to match the properties of our setup and attached as an extension to the open-source software 3D-Slicer [21]. Even though the therapy planning system (TPS) was calibrated for our setup, it can be adjusted to any other divergent X-ray microbeam source. Subsequently, we irradiated a mouse brain using MRT and visualized the resulting dose distribution by imaging histological sections of the brain stained for the DNA double-strand marker γ H2AX.

## 2. Materials and Methods

### 2.1. Microbeam Irradiation Setup

**Figure 1:**
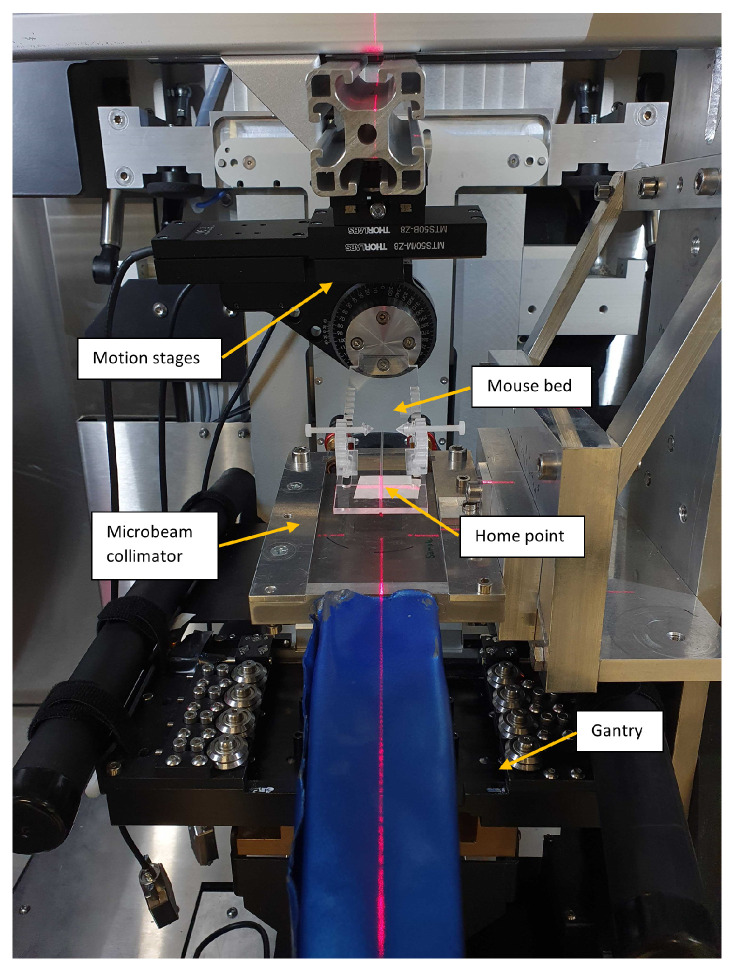
The image displays the microbeam setup within a small animal irradiator. The mice are placed and fixed on the mouse bed, which can be motioned perpendicular to the beam with motorized stages relative to the home point, defined as the middle of the radiation field and marked by the lasers.

As described before [20], the core component of the MRT setup is a 7 m m thick tungsten multislit-collimator, which was mounted at a source to collimator distance of 212 m m inside the small animal irradiator XenX and generates a 20 × 20 mm^2^ microbeam field (see Figure 1). The slit opening of the collimator is variable and was set to 30 μ m , whereas the ctc has a fixed value of 400 μ m . By attaching additional lead collimation, the microbeam field size can be reduced to either 10 × 10 mm^2^ or 5 × 5 mm^2^. To reduce the effective focal spot size seen by the collimator and thereby increase the peak-to-valley dose ratio, the collimator was tilted around the source by 8 ° . All irradiations were carried out with a 225 kVp X-ray spectrum filtered by 1 m m of aluminum.

To facilitate treatments of mice, a 3 mm thick PMMA mouse bed was produced in-house and attached to two motorized linear translation stages (MTS50-Z8 from Thorlabs Inc., Newton, MA, USA) and a motorized rotation stage (PRM1Z8 from Thorlabs), allowing 50 m m translation in both directions perpendicular to the beam and a 360 ° rotation. The position of the mouse bed in beam direction above the collimator was adjusted manually, and a minimal distance of 4 m m between the collimator and the mouse bed was achieved, limited by the design of the collimator holder. The mouse bed was tilted by 8 ° to match the tilt of the collimator. Further, two lasers were installed in the irradiation cabinet that met at the center of the irradiation field, defining a home point for animal positioning.

### 2.2. Development of the TPS

#### 2.2.1. Dose Calculation Utilizing a Hybrid Algorithm

Donzelli et al. [22] presented a hybrid dose calculation algorithm for synchrotron microbeam radiotherapy, which utilizes Monte Carlo (MC) methods and a kernel-based approach. In the first part, Monte Carlo simulations compute the dose deposited by photon interactions in each voxel, separating the dose originating from first-time interacting photons, $D_{primary}$ from the dose deposited by scattered photons, $D_{scatter}$ . Since the energy transfer mediated by electrons is entirely neglected in the MC part, macroscopic voxel sizes in the order of millimeters are sufficient to describe the photon dose distribution. In the second part of the hybrid algorithm, the energy transport by electrons in each voxel is calculated using convolution-based methods. Here, only electrons arising from primary photons are considered since they are distributed according to the photon fluence formed by the microbeam collimator and define the shape of the individual microbeams. The dose contribution from scattered photons is assumed to be equally distributed within a voxel. Thus, the resulting microbeam dose distribution in each voxel can be calculated using:

(1) $$D\left(x\right)=D_{primary}\;\cdot \;\left(\vartheta ⊛K_{el}^{1D}\right)\left(x\right)+D_{scatter}\;,$$

where *x* is the lateral position along the microbeam profile, ϑ is the normalized pattern of primary photon fluence given by the beam width and center-to-center distance of the microbeam field and $K_{el}^{1D}$ is the one-dimensional electron scatter kernel, for which a detailed derivation can be found in [23]. The ⊛ symbol denotes a convolution.

**Figure 2:**
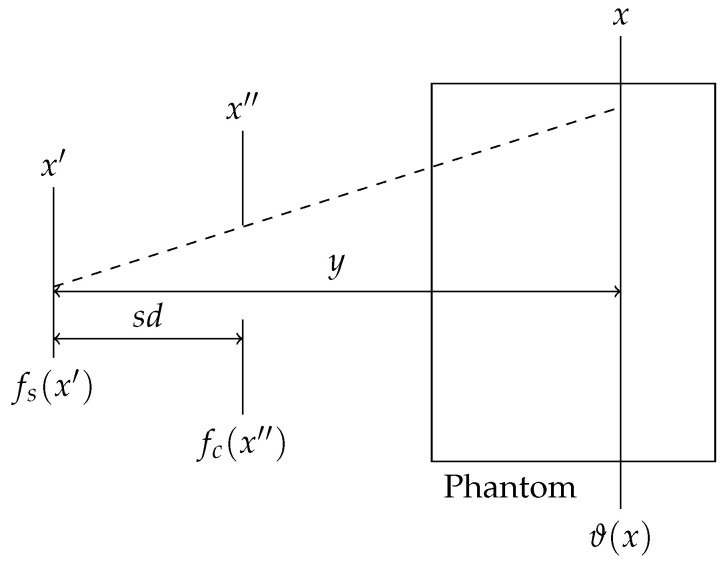
Sketch of the geometry of rays originating from a divergent source at $x^{\prime }$ , passing the collimator at the position $x^{\prime \prime }$ , which is placed at the distance sd from the source, hitting the phantom at a distance *y*. The resulting photon fluence is described by ϑ(x) . The functions $f_{s}\left(x^{\prime }\right)$ and $f_{c}\left(x^{\prime \prime }\right)$ describe the focal spot of the source and the collimator transmittance, respectively.

Since this algorithm was developed for quasi-parallel synchrotron radiation, it must be adapted for X-ray sources that produce divergent radiation. The pattern of the primary photon fluence is obtained from the ray geometry between the source, collimator, and target as shown in Figure 2, which can be calculated using the following integral:

(2) $$\vartheta \left(x\right)=\int f_{s}\left(x^{\prime }\right)\;f_{c}\left(x^{\prime \prime }\left(x,x^{\prime }\right)\right)\;dx^{\prime }\;,$$

where $f_{s}\left(x^{\prime }\right)$ and $f_{c}\left(x^{\prime \prime }\left(x,x^{\prime }\right)\right)$ describe the focal spot as the X-ray source and the collimator transmittance, respectively. Inserting Equation (2) into (1) leads to:

(3) $$D\left(x\right)=D_{primary}\;\cdot \;\left(f_{c}^{\prime }⊛\left(K_{el}^{1D}⊛f_{s}^{\prime }\right)\right)\left(x\right)+D_{scatter}\;,$$

where the modified source and collimator functions $f_{s}^{\prime }$ and $f_{c}^{\prime }$ are given by:

(4) $$\begin{array}{cc} f_{s}^{\prime }\left(x\right) & =f_{s}\left(\frac{1}{1-\frac{y}{sd}}\;x\right),\\ f_{c}^{\prime }\left(x\right) & =f_{c}\left(\frac{sd}{y}\;x\right). \end{array}$$

Equation (3) is valid for any divergent source modulated by any randomly shaped collimator. Therefore, the algorithm can also be applied to other sources such as the line focus X-ray tube [24]. However, for the XenX source, we modeled the focal spot with a Gaussian function:

(5) $$f_{s}\left(x\right)=\frac{1}{\sqrt{2\pi \sigma }}e^{-\frac{x^{2}}{2\sigma^{2}}},$$

where σ is the standard deviation and is determined experimentally.

#### 2.2.2. TPS Module in 3D-Slicer

We developed a graphical user interface as an extension to 3D-Slicer, which is an open-source software for the visualization and analysis of medical image data sets [21], and coupled it to the dose calculation engine. The therapy planning module allows to load a planning computed tomography (CT) image and to define the field size and the position of the isocenter. Further, the beam, which by default is centered around the isocenter, can be translated and rotated by means of the collimator-, couch-, and gantry angles. Thereafter, the dose calculation using the hybrid algorithm can be triggered. The geometry for dose calculation is based on the CT image, and the material composition in each voxel is reconstructed from its Hounsfield unit [22,25]. The resulting dose distribution is visualized as an overlay onto the CT image. Furthermore, the micrometer-sized dose profiles within a specific voxel can be calculated and visualized.

#### 2.2.3. Calibration of the Dose Calculation Algorithm

In a calibration process of the dose calculation engine, we adjusted the focal spot width σ such that calculated and measured dose profiles matched. The peak doses, valley doses, and dose profiles were assessed in a 100 × 54 × 54 mm^3^ PMMA phantom at different depths using GafChromic™ EBT3 (Ashland, Wilmington, DE, USA) films. The entire 20 × 20 mm^2^ microbeam field was used for the irradiation. The same phantom and field were defined in the hybrid algorithm, and the resulting doses were compared against the experimental results.

For quantitative dosimetry, the films were calibrated with uniform doses in a RS225 X-ray irradiator (Xstrahl, Suwanee, GA, USA). Its dose rate was measured using a Farmer TM30010 ionization chamber (PTW Freiburg GmbH, Freiburg, Germany), and six different films were irradiated with a dose in the range of (0–10) Gy. After irradiation, the films were scanned with a Reflecta ProScan 10T (Reflecta GmbH, Eutingen, Germany) film scanner, and their gray values were correlated to the irradiated dose by fitting the data points with:

(6) $$D\left(gv\right)=a+\frac{b}{gv+c}\;,$$

where *D* is the dose, gv is the grey value and *a*, *b*, and *c* are the fit parameters. More details about film handling are provided in [26].

#### 2.2.4. Validation of the TPS

A phantom study was performed to validate the TPS. For this, a 20 × 40 × 20 mm^3^ PMMA phantom was placed on the mouse bed and exposed to different microbeam fields. The peak- and valley dose rates were assessed at different depths in the phantom with film dosimetry. For dose calculation with the TPS module in 3D-Slicer, a CT image of the phantom was acquired, where the films were placed inside the phantom at the same position as for the radiation exposure. The beam geometry in the planning module was set to match the experimental conditions, and the dose distribution obtained by the TPS was compared with the measured results.

### 2.3. Workflow for Animal Treatment

#### 2.3.1. CT Image Acquisition

**Figure 3:**
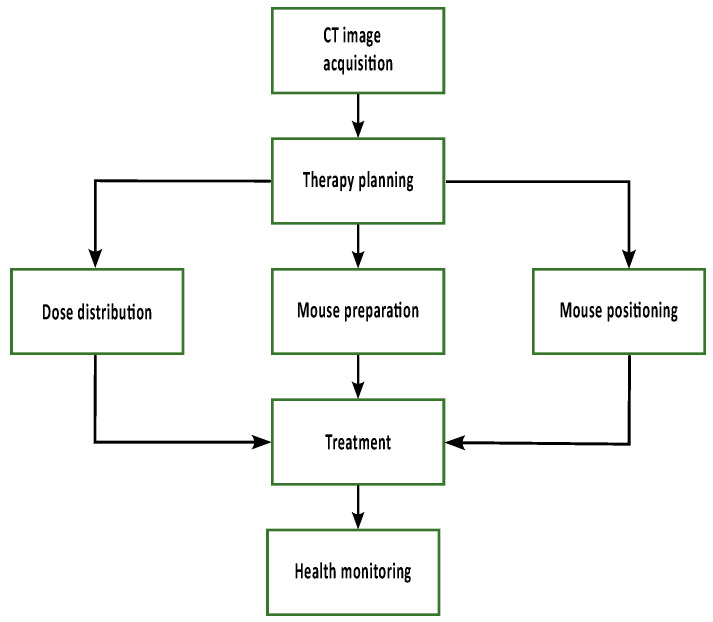
At first, a CT image of the animal is acquired and used for therapy planning. Irradiation parameters, such as field size and beam direction, can be chosen and optimized to ensure target volume coverage. The TPS provides the dose distribution to calculate the irradiation time and the parameters to position the mouse in the field. In preparation for treatment, the animals are injected with glucose, and an eye cream is applied. During treatment, the mice are anesthetized using 2% Isoflurane, and their breathing is observed and regulated by adjusting the isoflurane concentration during treatment. After irradiation, a health check is performed.

As illustrated in Figure 3 the first step for animal treatment is the acquisition of a CT image, which was obtained using the nanoScan SPECT/CT (Mediso GmbH, Münster, Germany) with a peak tube voltage of 70 kVp and a current of 270 μ A . During CT image acquisition, the mouse was placed on the identical mouse bed that was used for treatment and fixed with ear screws.

#### 2.3.2. Therapy Planning

The 3D-Slicer module described in Section 2.2.2 was used for therapy planning. After loading the CT image of the mouse into the planning system, the isocenter was set to the reference point engraved on the mouse bed, which was visible in the CT image. After the field size was selected, the beam was positioned such that it covered the target volume. The shift of the beam with respect to the isocenter directly corresponded to the alignment of the mouse in the radiation field described in the following section. Thereby, the TPS provided the mean peak and valley doses, the microbeam dose profile in the target volume, and the parameters for positioning.

#### 2.3.3. Mouse Irradiation

C57BL/6J mice (Charles River, Sulzfeld, Germany) were kept in the animal care facility at Helmholtz Centre Munich following the German Animal Welfare Policy after approval by the government of Upper Bavaria (ROB-55.2-2532.Vet_02-20-39). Before treatment, the health of the mice was checked, glucose fluid was injected subcutaneously, and Bepanthen^®^ (Bayer AG, Berlin, Germany) eye cream was applied to the eyes to prevent dehydration. The mice were anesthetized with 2% isoflurane, and their breathing was monitored throughout the treatment using a webcam. The isofluran concentration was adapted depending on the breathing frequency, aiming for 1 Hz . Furthermore, the temperature within the XenX was held around 28 °C to prevent hypothermia during anesthesia. To position the mouse according to the treatment plan, two lasers were installed in the XenX cabinet that marked the center of the radiation field. Subsequently, the animal bed was positioned such that the cross engraved in it matched the position marked by the lasers. From there, the position was adjusted according to the parameters provided by the TPS. We irradiated half of the brain with 20 Gy peak dose, according to the treatment plan presented in Section 3.3. Following the irradiation, the animals were under observation until they regained consciousness from anesthesia, and they were monitored until their behavior returned to normal.

#### 2.3.4. Brain Excision

To excise the mouse brain, a transversal incision into the skin was made at the dorsal region of the neck, directly behind the occipital bone. This skin incision was extended by a sagittal cut from the foramen magnum towards the parietal region to expose the skull. Using bone scissors, a sagittal cut in the skull was made starting from the foramen magnum along the external occipital crest through the occipital bone and along the sagittal suture towards the parietal bone. The two halves of the skull were carefully separated using forceps to reveal the brain. With the help of the forceps, the intact brain was separated from the remaining underlying tissue, removed, fixed, and stored in formalin.

#### 2.3.5. Tissue Sectioning, Antibody Staining, and Signal quantification

Brain tissue specimens were fixed in 4% (*w*/*v)* neutrally buffered formalin and embedded in paraffin. For γ H2AX immunoreactivity, 3 μ m tissue sections were stained on a Discovery Ultra automated stainer (Ventana Medical Systems, Tucson, AZ, USA) using rabbit anti- γ H2AX antibody (1:400; #2577S, Cell Signaling, Danvers, MA, USA) and a biotinylated secondary antibody (1:750, BA-1000, Vector Laboratories Inc., Burlingame, CA, USA). Signal detection was performed using the Discovery™ DABMap™ Kit (Ventana Medical Systems, Tucson, AZ, USA). The stained tissue sections were scanned with an Axio Scan.Z1 digital slide scanner (Zeiss, Jena, Germany) equipped with a 20× magnification objective and quantified by using the commercially available software Definiens Developer XD™ 2 (Biocompare, San Francisco, CA, USA). Within the manually annotated regions of irradiated and unirradiated tissue, the mean chromogen brown intensity of detected nuclei was calculated.

## 3. Results

### 3.1. Calibration of the Dose Calculation Algorithm

Experimental and simulated peak doses, valley doses, and dose profiles agreed best with a model of the focal spot of the XenX that used a Gaussian function with a standard deviation of σ=0.58 mm . When defining the focal spot size as the full width of the Gaussian at 10% of its maximum intensity, according to the DIN norm EN 12543-1 [27], the focal spot size *d* can be calculated as:

(7) $$d=\sqrt{\sigma^{2}\;8\;ln\left(10\right)}.$$

**Figure 4:**
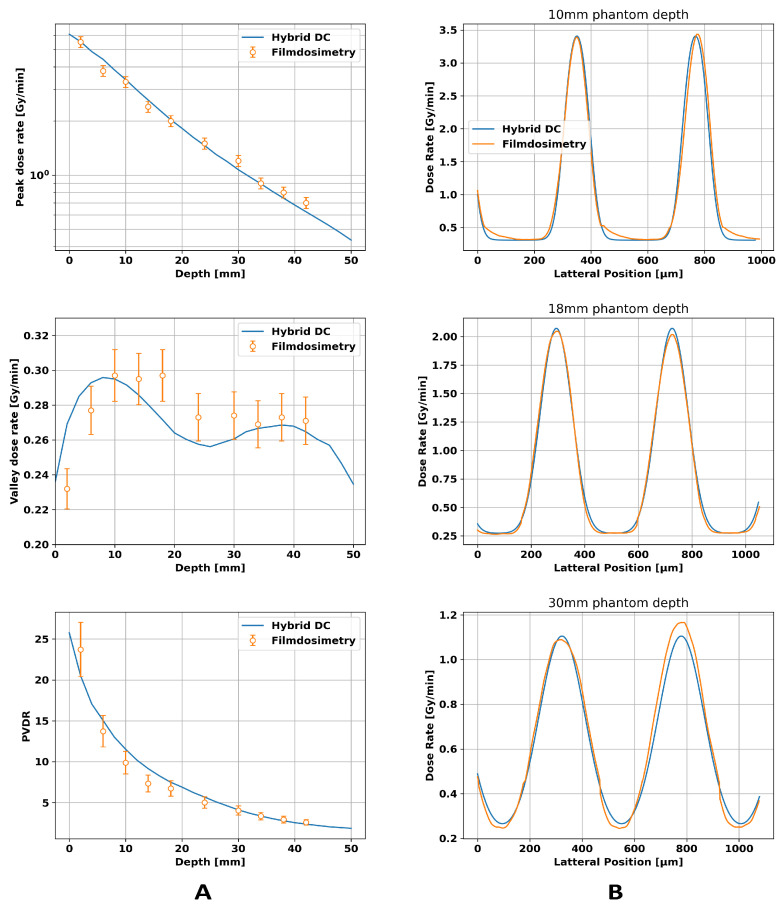
Comparison between film dosimetry and the hybrid dose calculation algorithm for a 20 × 20 mm^2^ microbeam field hitting a 100 × 54 × 54 mm^3^ PMMA phantom. Column (**A**) displays the depth-dependent values for peak dose, valley dose, and peak-to-valley dose ratio (PVDR), while column (**B**) presents the dose profiles at different depths.

Inserting the value of σ into Equation (7) yields a focal spot size of 2.5 m m . According to an internal test by the manufacturer, the focal spot size of the XenX is 3.55 × 2.95 mm^2^. The observed reduction of the focal spot size can be explained by the collimator tilt, which leads to a reduced projection of the focal spot. Applying the value of σ in the dose calculation engine led to the results illustrated in Figure 4. While the peak dose decreased nearly linearly on a logarithmic scale, which was expected according to Beers law, the valley dose showed two local maxima. The first maximum can be attributed to a build-up effect caused by scattered photons. In contrast, the second maximum at a depth of 38 mm arose due to the divergence of the beam, which led to the overlapping of adjacent beam penumbras, causing a rise of the valley dose. Benchmarking the hybrid dose calculation algorithm against film dosimetry showed a relative difference of ≤8% for the peak dose and the valley dose. The dose profiles showed an increase in the FWHM (full width at half maximum) and in the center-to-center distance with increasing depth for both film dosimetry and the hybrid algorithm, which is expected due to the beam divergence and agreed within an error of 10% considering deviations in dose rate and the FWHM.

### 3.2. Validation of TPS

**Figure 5:**
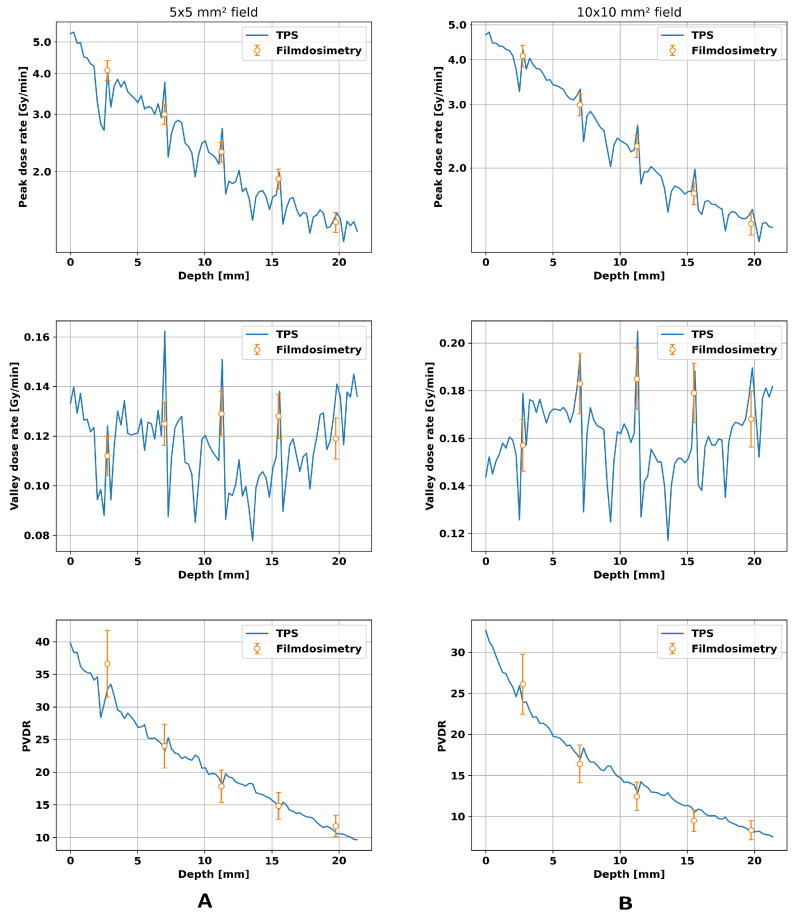
Comparison of film dosimetry with the TPS for a 5 × 5 mm^2^ field in the column (**A**) and for a 10 × 10 mm^2^ field in column (**B**). Both fields hit a 20 × 10 × 20 mm^3^ PMMA phantom placed on the mouse bed, and in both cases, the peak- and valley dose rates and the PVDRs were compared. For dose calculation, a CT image of the phantom with the EBT3 films inside was acquired and fed into the algorithm. The local fluctuations in the calculated dose rate by the TPS correspond to the position of the film in the phantom. The peak-to-valley dose ratio is abbreviated as PVDR.

The phantom study verified the parameters found in the previous section. The results are presented in Figure 5. All dose rate plots show local increases at the position of the films. The peak dose rates show similar values for the 5 × 5 mm^2^ and the 10 × 10 mm^2^ field and a linear decrease over depth on a logarithmic scale. The valley dose rates, however, are higher in the larger field since a larger field inherently has a higher number of scattered X-rays, leading to higher valley doses. For the smaller field, the experimental data agreed with the predicted doses for peak dose rate, valley dose rate, and PVDR within an error of ≤9%, while the larger microbeam field showed a relative difference of ≤ 8 % between the film and dose calculation.

### 3.3. Therapy Plan for a Mouse Treatment

**Figure 6:**
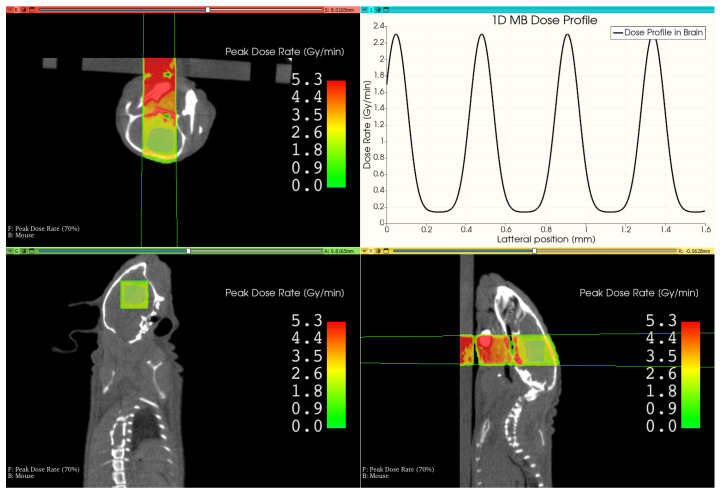
This image shows the resulting view of the developed MRT therapy planning system in 3D-Slicer. It shows different views of the mouse CT, overlaid by the peak dose distribution and the outline of the beam. The upper right image shows the lateral microbeam dose profile inside the brain.

Figure 6 shows the resulting treatment plan for the mouse irradiation. The beam, which is outlined in green in the images, penetrates through the mouse bed and the lower part of the mouse head before reaching the brain. The resulting peak dose rate is shown as an overlay to the CT image, which decreases with depth in the mouse but locally rises in denser material like bone. By computing the mean dose rate values in the targeted brain area, the peak dose rate was found to be 2.3 Gy min^−1^, and the valley dose rate was 0.14 Gy min^−1^, leading to a mean PVDR of 16 with the FWHM of the individual microbeams being 138 μ m separated by a ctc of 430 μ m . Since we targeted for a peak dose of 20 Gy in the brain, the mouse was irradiated for 522 s .

**Figure 7:**
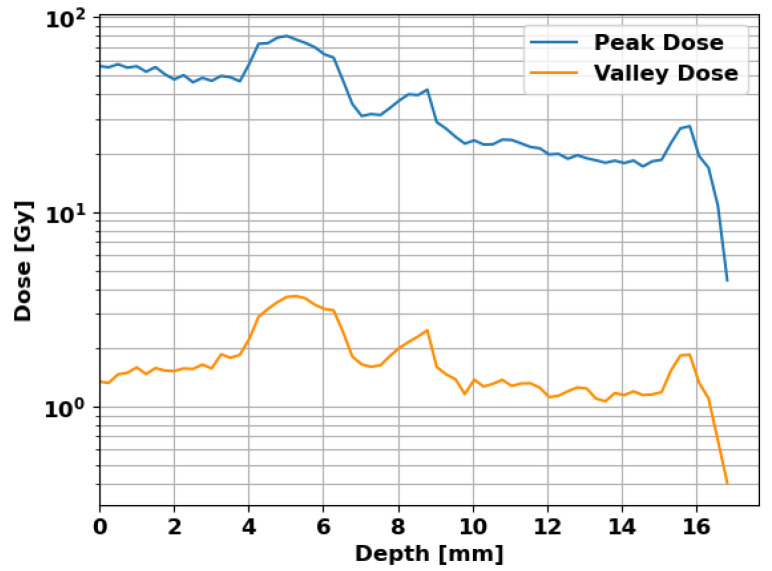
Peak and valley doses in the mouse with depth in the beam direction. The targeted brain is located at a depth between 9 m m and 15 m m , receiving a peak dose of 20 Gy and a valley dose of 1.2 Gy .

Figure 7 shows the peak and valley doses against depth in the mouse in the beam direction. The peak dose shows a decreasing trend over depth with local rises in the bone, which are observed in the valley dose as well. For the targeted brain area at a depth between 9 m m and 15 m m , the peak dose is 20 Gy as intended and the valley dose is 1.2 Gy .

### 3.4. Histological Section of an Irradiated brain

**Figure 8:**
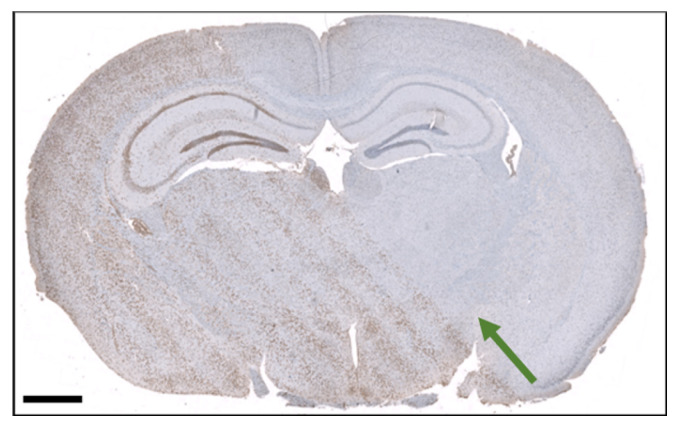
Section of the mouse brain after microbeam irradiation and stained with γ H2AX labeling. The scale bar is 1 mm. The green arrow indicates the beam direction.

Figure 8 shows a histological section of the irradiated mouse brain. The γ H2AX distinguishes peak and valley regions, and the microbeam pattern is clearly visible. The quantification of the signal using the Definiens Developer XD™ 2 (Biocompare, San Francisco, CA, USA) software confirms the higher intensity of γ H2AX signal in peak regions with an intensity of 0.62±0.28 compared to 0.44±0.19 in valley regions, where we analyzed 14,881 cells in the peak region and 11,309 cells in the valley region. Further, the image allows to determine that the center-to-center distance increases by around 3% from the beam entrance to exit in the brain. Given our radiation geometry with a source-to-collimator distance of 212 mm , the factor *x* by which the ctc increases can be calculated using the theorem of intersecting lines. With the thickness of the brain in the beam direction being 6 m m , this leads to:

(8) $$x=\frac{212\;mm+6\;mm}{212\;mm}=1.03.$$

Thereby, the calculated and measured increases in the ctc match.

## 4. Discussion

In this work, we demonstrated for the first time that mice can be treated with microbeams using a conventional small animal irradiator. We adjusted an existing dose calculation algorithm that was developed assuming quasi-parallel microbeams generated by synchrotrons to be able to calculate the dose deposited by microbeams generated by any divergent source. Further, we calibrated this algorithm to our specific source and setup and attached it to 3D-Slicer. This resulted in a comprehensive therapy planning system with a graphical user interface. The functionality of the TPS was then successfully verified with a phantom study that mimicked the circumstances of an animal treatment. Afterward, a mouse was taken through every step for treatment planning and then successfully treated with microbeams using our developed setup. Despite the proven feasibility of the microbeam treatment with our setup, it does have some limitations.

One limitation of the presented setup is that the beam divergence that caused the microbeams to widen significantly, which restricted the FWHM of the peaks to 140 μ m in the mouse brain after minimizing the distance of the brain to the collimator. The preferred peak width of 50 μ m could not be reached with this setup.

The second limitation is the low dose rate, which may influence the spatial fractionation due to target movement and lead to long irradiation times. Since we achieved a valley dose rate of 014 Gy min^−1^, irradiations aiming for 10 Gy in the valley would last more than an hour, making treatments with considerably higher valley doses very time consuming. For extended exposure times, repair mechanisms will play an important role and have to be taken into account when evaluating biological data [28,29]. The Lea–Catcheside factor *G* in the linear quadratic model presents a simple way to approximate the effect of protracted exposures in radiobiological modeling. Assuming a mono-exponential reciprocal repair model with the repair rate λ and an irradiation at constant dose rate for irradiation time *T*, *G* can be calculated by [30]:

(9) $$G=\frac{2}{\lambda T}\left(1-\frac{1-e^{-\lambda T}}{\lambda T}\right)\;.$$

For late-responding tissue, a repair rate of λ = 0.5 Gy h^−1^ is typically assumed [31], which leads to G=0.85 at an exposure time of one hour. Assuming $\frac{\alpha }{\beta }=2\;\mathrm{Gy}$ , a dose of $D_{protracted}=10\;\mathrm{Gy}$ , given within one hour, corresponds to a single-fraction short exposure with a dose of:

(10) $$D_{short}=\frac{1}{2}\frac{\alpha }{\beta }+\sqrt{\frac{1}{4}{\left(\frac{\alpha }{\beta }\right)}^{2}-\frac{\alpha }{\beta }D_{protracted}+GD_{protracted}^{2}}=9.1\;\mathrm{Gy}\;.$$

Apart from the repair mechanisms, the long exposure times may also affect the spatial dose modulation due to organ motion. The results shown in Figure 8 demonstrate preservation of the spatial dose modulation, with the observed beam divergence being in line with the expected outcome. However, the data did not allow further conclusions on other parameters like PVDR, FWHM, or absolute dose values since quantitative analysis of the tissue sections is limited. Quantitative γ H2AX measurements showed a high background signal, even though high-dose regions could be clearly distinguished from low-dose regions.

Further, we demonstrated that the hybrid dose calculation algorithm, originally designed for MRT with highly parallel synchrotron radiation, can be modified and applied to MRT at divergent X-ray sources. This adaptation enables the calculation of doses for microbeams generated using the conventional X-ray irradiator XenX. Although the algorithm is optimized for the XenX, its core is also applicable to microbeams generated at other divergent X-ray sources. The predicted doses with the TPS agree with the film dosimetry measurements with an accuracy of better than 10%. Accuracy criteria for microbeam radiation treatments were worked out by Martínez-Rovira et al. [32], specifying that discrepancies between the experimental data and therapy planning should ideally be below 3% for clinical application. Despite not meeting clinical precision, our treatment planning system demonstrates promising utility for pre-clinical investigations. This system provides a valuable platform for research in MRT, potentially advancing the understanding of its mechanisms.

Moreover, the challenges posed by the low dose rate can be mitigated by employing tumor models that are less affected by organ motion. In this context, there are potential applications for subcutaneous tumor models in the leg or orthotopic tumors in the brain. These models offer controlled conditions that can aid in studying the effects of microbeam radiation therapy.

## 5. Conclusions

In this work, we demonstrated the feasibility of MRT in vivo exposures at a compact small animal irradiator and showed that a microbeam pattern was maintained in histological sections of a mouse brain. We adapted a hybrid dose calculation algorithm, initially designed for synchrotron microbeam radiation, to calculate the dose of microbeams generated by a conventional small animal irradiator. Additionally, we successfully integrated this algorithm into 3D-Slicer, resulting in a comprehensive TPS with a dose calculation accuracy of better than 10%. Thus, we present a framework capable of facilitating pre-clinical research in MRT. For clinical applications, other X-ray sources with considerably higher dose rates and improvements in treatment planning accuracy are required.

## Data Availability

Data are contained within the article.

## Abbreviations

The following abbreviations are used in this manuscript:

MRT	Microbeam radiation therapy
DNA	Deoxyribonucleic acid
CTC	Center-to-center distance
PVDR	Peak-to-valley dose ratio
TPS	Therapy planning system
MC	Monte Carlo
CT	Computed tomography
SPECT	Single photon emission computed tomography
*w*/*v*	Weight by volume
FWHM	Full width at half maximum
